# *Dientamoeba fragilis* in Ulcerative Colitis: Analysis of Clinical Findings and Biochemical Parameters

**DOI:** 10.3390/pathogens14070674

**Published:** 2025-07-09

**Authors:** Ismail Taskiran, Erdogan Malatyali, Ibrahim Yildiz, Levent Durmus Guler, Evren Tileklioglu, Hatice Ertabaklar, Sema Ertug

**Affiliations:** 1 Division of Gastroenterology and Hepatology, Department of Internal Medicine, Faculty of Medicine, Aydin Adnan Menderes University, 09010 Aydin, Turkey; itaskiran@adu.edu.tr (I.T.); durmus_guler@hotmail.com (L.D.G.); 2 Department of Parasitology, Faculty of Medicine, Aydin Adnan Menderes University, 09010 Aydin, Turkey; dr.ibrahimyildiz@gmail.com (I.Y.); evren.tileklioglu@adu.edu.tr (E.T.); hatice@adu.edu.tr (H.E.); sertug@adu.edu.tr (S.E.)

**Keywords:** *Dientamoeba fragilis*, ulcerative colitis, frequency, polymerase chain reaction, genotype, biochemical parameters, symptoms, treatment response

## Abstract

Although *Dientamoeba fragilis* is a common protozoan in humans, its pathogenicity and clinical significance in human diseases remain poorly understood. This study aimed to determine the frequency of *D. fragilis* in adult ulcerative colitis patients and to assess its relationship with clinical findings, disease characteristics, and biochemical parameters. Patient data were analysed in a prospective, single-centre, cross-sectional design. Faecal samples were consecutively collected from June to December 2024 and screened for *D. fragilis* positivity using polymerase chain reaction. Of the 110 patients, 33 (30%) were in the active stage of the disease, while 77 (70%) were in remission. The overall frequency of *D. fragilis* was 10.9% (*n* = 12), with all isolates classified as genotype 1 according to SSU rRNA sequence analysis. Other protozoa identified were *Blastocystis* sp. (*n* = 5, 4.5%), *Entamoeba coli* (*n* = 1, 0.9%), and *Iodamoeba bütschlii* (*n* = 1, 0.9%). Two patients were co-infected with *D. fragilis* and *Blastocystis*. No significant associations were found between *D. fragilis* positivity and the disease stage, gastrointestinal symptoms, treatment response, or biochemical findings. In conclusion, despite the relatively small sample size, these findings highlight a limited clinical role of *D. fragilis* in adult ulcerative colitis patients.

## 1. Introduction

*Dientamoeba fragilis* was first described in 1918 as a binucleate member of intestinal amoebas. It was named “fragilis”, as the organism failed to maintain its morphological integrity outside the host. Ultrastructural studies, along with fluorescent antibody labelling and DNA-based analyses, have confirmed that it is phylogenetically closer to trichomonads rather than to amoebas [1]. Trophozoites are small (5–15 µm), are pleomorphic, and move with broad pseudopodia, often difficult to observe without staining. The majority are bi-nucleated, though single-nucleus forms are also common (20%). A finely granular and vacuolated cytoplasm is typical, often containing ingested bacteria, yeasts, and other debris from its phagocytic feeding activity. Transmission via helminth eggs was originally proposed in the life cycle of *D. fragilis*. However, the characterisation of cyst and precystic forms in human clinical samples has suggested a possible mode of transmission, even in the absence of nematode eggs [2]. *D. fragilis* cysts are generally oval to round and are smaller than trophozoites (5 to 8 µm). In addition, hydrogenosomes, endoplasmic reticulum, pelta-axostyle structures, costa, axonemes and nuclei are present in the cysts [3]. *D. fragilis* is prevalent in both developed and undeveloped countries, despite methodological variations in reported prevalence, ranging from 0.2% to 91% [4,5]. Risk factors for *D. fragilis* infection are generally consistent with faecal–oral-transmitted parasites, including poor personal hygiene, living in rural areas, and co-infection with pinworms [6,7]. In addition, the high frequencies in developed countries are closely related to the use of molecular-based methods [8]. The major challenges for diagnostic laboratories are standardisation and the limited sensitivity of microscopy, resulting from the small size and fragility of the organism. Molecular-based methods, particularly quantitative-PCR (qPCR), show superior sensitivity over microscopy and xenic cultures in the diagnosis [9]. In addition, these techniques allow the genetic characterisation of *D. fragilis* isolates. The amount of molecular epidemiological research has significantly increased in the past decade [10]. Interestingly, the number of polymorphisms was low in previously tested genetic markers for *D. fragilis* including small subunit ribosomal (SSU rDNA), housekeeping genes, *EF1α*, and *actin* [11,12]. Of the two well-known genotypes of *D. fragilis*, genotype 1 is widely prevalent in human faecal samples on a global scale. Gorillas, pigs, cats, dogs, cattle, and budgerigars were also defined as the carriers of this genotype [13,14,15]. Also known as the Bi/PA strain, genotype 2 has only occasionally been reported in molecular epidemiological studies. It remains mainly uncertain whether *D. fragilis* causes significant symptoms in humans. Despite its widespread presence in asymptomatic individuals, *D. fragilis* infection has been linked to some gastrointestinal symptoms, particularly diarrhoea and abdominal pain [16,17]. It was reported that a single-dose secnidazole treatment eradicated *D. fragilis* in 34 patients (97.1%); however, 1 patient required an additional dose. After the eradication of *D. fragilis*, symptoms resolved in 27 cases (77.1%) and improved in 8 cases (22.9%) [18]. Treatment of *D. fragilis* is generally recommended when symptoms are present and no other pathogens are detected in clinical specimens [4].

Ulcerative colitis (UC) is a chronic, recurrent inflammatory disease of the colon characterised by inflammation limited to the mucosal layer. In active disease, the most prominent symptom is bloody, mucus-filled diarrhoea. Aminosalicylates (5-ASA, mesalamine) are the first-line therapy for mild-to-moderate ulcerative colitis. In patients with an inadequate response to 5-ASA alone, combination therapy with azathioprine (AZA) is commonly applied. For patients who do not respond to conventional therapies, biological agents are introduced as advanced treatment options. Corticosteroids are also used for the short-term management of acute flares to achieve rapid reduction in inflammation [19]. The current estimated prevalence of UC is 5 million cases, and the incidence tends to increase worldwide [20]. The coexisting gastrointestinal infections can both exacerbate the disease and limit the effectiveness of these medications. Intestinal microbiota imbalances, or dysbiosis, may also play a significant role in disease activation [21]. Most microbiota studies in UC have focused on bacteria, but parasitic agents have been investigated in only a limited number of studies. They may contribute to inflammation and the activation of the colonic mucosa, or conversely, they may limit inflammation through immunomodulatory effects [22,23,24]. The objectives of this study were to determine the prevalence and genotypic variations in *D. fragilis* in adult UC patients and to evaluate its clinical significance in relation to disease stage, duration, symptoms, treatment response, and biochemical parameters.

## 2. Materials and Methods

### 2.1. Study Design

This study was designed as a prospective, single-centred, and cross-sectional observational study. Adult UC patients, both newly diagnosed and those in follow-up in Aydin Adnan Menderes University Hospital between June and December 2024, were included in the study. Aydin, situated in south-western Turkey (37°50′53″ N, 27°50′43″ E), has a Mediterranean climate with hot, dry summers and mild, wet winters. Its economy is primarily driven by agriculture, tourism, and trade. This study was reviewed and approved by the local ethics committee (Approval No: 2024/96). Informed consent was obtained from all patients.

### 2.2. Patients and Faecal Samples

The criteria for the diagnosis of adult UC included (i) evidence from colonoscopy, (ii) results from pathological examination of biopsies, and (iii) relevant clinical findings. Disease activity was determined according to the Mayo score [25]. A Mayo score of 0–1 indicated remission, while a score of 2–3 suggested active disease. The Montreal classification was used to classify the colonic involvement and phenotypes of the patients [26]. 5-ASA was the first-line therapy in our clinic for the treatment of UC patients. If there was an inadequate response to monotherapy, AZA was added as combination therapy. For patients who did not respond adequately to conventional therapies (5-ASA, AZA, and corticosteroids for flares), biological agents were used to achieve or maintain remission. The criteria for treatment response included relief of symptoms and endoscopic healing. Patients meeting these criteria were classified as responders, while those who did not were classified as non-responders to treatment. A single faecal sample was collected from each patient and examined using direct microscopy of wet-mount preparations. Following routine coprological examination, the samples were stored at −20 °C until molecular analyses.

### 2.3. Determination of D. fragilis Positivity

Genomic DNA was extracted directly from frozen faecal samples using a commercial kit (Qiagen GmbH, Hilden, Germany). *D. fragilis* positivity was detected with polymerase chain reaction (PCR)-based amplification of an approximately 863 bp fragment of the SSU rRNA gene using primers DF400 and DF1250. The reaction was prepared in a 30 µL:1 µL of template DNA, 1 U of Taq DNA polymerase, 0.2 mM of dNTPs, 0.4 µM of each primer, 2 mM of MgCl_2_, and PCR buffer with (NH_4_)_2_SO_4_. Thermal cycling conditions were set as follows: initial denaturation at 94 °C for 3 min, 30 cycles (94 °C for 1 min, 57 °C for 1.5 min, and 72 °C for 2 min), and final extension at 72 °C for 7 min. The amplicons were analysed by electrophoresis on a 1.5% agarose gel and visualised using a UV imaging system (Vilber Lourmat, Collégien, France). Genomic DNA of a previous *D. fragilis* isolate, validated by partial 18S rRNA sequencing along with several housekeeping genes, was included as a positive control for this study [27].

### 2.4. Determination of Genotypes

The positive amplicons following electrophoresis were sequenced by a commercial facility (Medsantek Co., Ltd., Istanbul, Turkey). The sequences were edited and subsequently aligned via the ClustalW algorithm in BioEdit software, version 7.7.1 (Ibis Biosciences, Carlsbad, CA, USA). A phylogenetic tree was created according to the evolutionary distances of our isolates, references, and outgroups. The distances were calculated using the Maximum Composite Likelihood method. After a BLAST software, version 2.16.0 (NCBI, Bethesda, MD, USA) search in Genbank, *D. fragilis* partial SSU rRNA gene sequences from Turkey, Italy, Germany, Australia, Iran, and the Czech Republic were included in the phylogenetic analysis. The references were publicly available nucleotide sequences of genotype 1 (AY730405) and genotype 2 (U37461). The partial trichomonad SSU rRNA sequences from other hosts such as *Histomonas meleagridis* (host: avian species), *Tritrichomonas foetus* (host: cattle), and *T. nonconforma* (host: lizard) were also included in the analysis as outgroups. Evolutionary relationships of taxa were determined using the Neighbor-Joining method, with bootstrap values (based on 1000 replicates) in MEGA software, version 11.0.13 [28].

### 2.5. Analysis of Clinical Findings and Biochemical Parameters

Before providing stool samples, each patient was received a physical examination, and their medical history was recorded. During the anamnesis, patients were questioned about faecal consistency, the frequency of diarrhoea (if present), the presence of bleeding or mucus in faeces, abdominopelvic pain, and fatigue. The erythrocyte sedimentation rate (ESR), C-reactive protein, aspartate aminotransferase (AST) level, alanine aminotransferase (ALT) level, creatinine level, total leukocyte count (TLC), neutrophil count, eosinophil count, and iron deficiency anaemia (IDA) of patients were analysed in the present study. These parameters were among the most frequently requested routine tests in the hospital information system. The reference ranges for ESR were as follows: males under 50 years: 0–15 mm/h; males 50 years or older: 0–20 mm/h; females under 50 years: 0–20 mm/h; and females 50 years or older: 0–30 mm/h. The cut-off value for C-reactive protein was 5 mg/L. Reference ranges for liver enzymes were 5–34 U/L for AST and 0–55 U/L for ALT. Among haematological parameters, the normal range of TLC was 4–10 × 10^3^/µL, the neutrophil count was 1.5–6 × 10^3^/µL, and the eosinophil count was 0.04–0.36 × 10^3^/µL.

### 2.6. Statistical Analyses

The relationships between *D. fragilis* positivity and clinical, biochemical, and disease-related variables were evaluated using statistical methods. Fisher’s exact test (only for 2 × 2 tables) and Student’s T-test were used to analyse the categorical (abdominal/pelvic pain, rectal bleeding, diarrhoea, weight loss, fatigue, nausea/vomiting, treatment response, disease stage, ESR, C-reactive protein, AST, ALT, neutrophil count, eosinophil count, and iron deficiency anaemia) and continuous variables (disease duration and body mass index), respectively. Due to the limited sample size, multivariate analyses were not performed. An alpha level of 0.05 was used to determine statistical significance. All analyses were conducted with Statistical Package for the Social Sciences, SPSS vs. 26 (IBM, Armonk, NY, USA).

## 3. Results

### 3.1. D. fragilis Positivity and Genotypes

**Figure 1:**
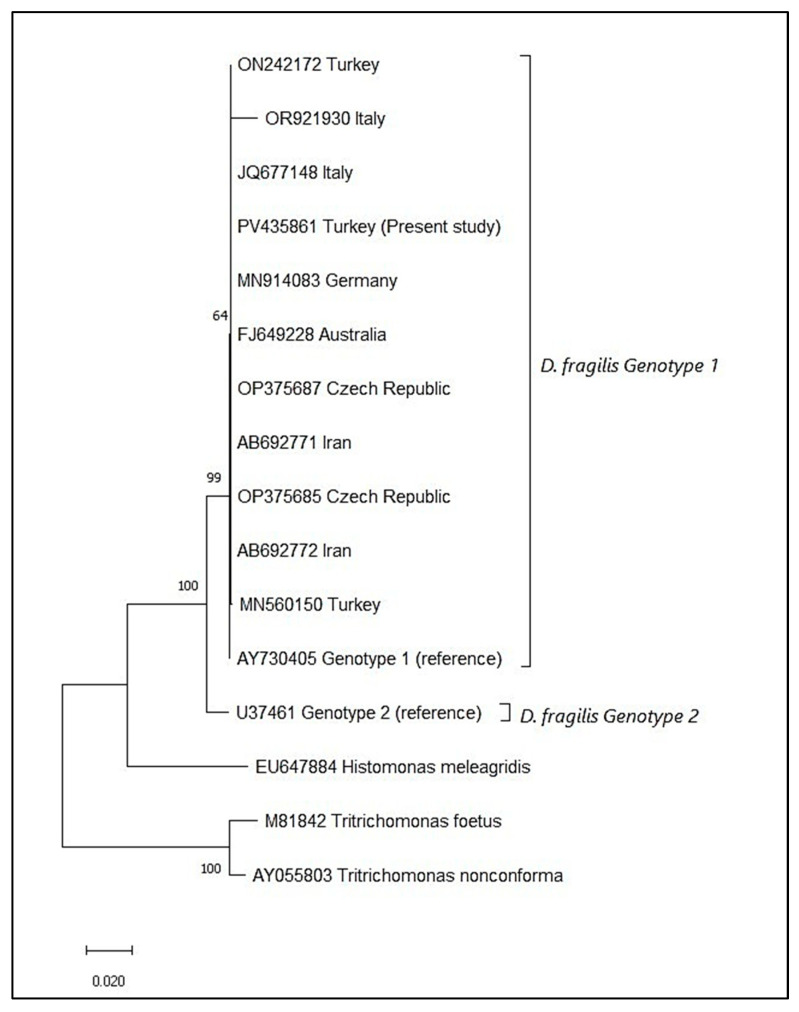
Phylogenetic relationship between *D. fragilis* isolates and references.

Faecal samples from 110 adult ulcerative colitis patients were collected during the study period. The overall positivity rate of *D. fragilis* was 10.9% (*n* = 12) based on PCR analysis. *Blastocystis* sp. was detected in five patients (4.5%) following direct microscopy of wet-mount preparations. One patient was positive for *Entamoeba coli* cysts, and another patient was positive for *Iodamoeba bütschlii* cysts, each representing 0.9% of the study population. Two (16.6%) of *D. fragilis*-positive patients additionally harboured *Blastocystis* sp. in faecal samples. The partial SSU-rRNA gene sequences of 12 samples in the study were identical to each other and submitted to Genbank (Acc. No. PV435861). All the isolates were defined as genotype 1 after phylogenetic analysis. The evolutionary relationships of the isolates with reference sequences and outgroups are presented in Figure 1.

### 3.2. The Analysis of Clinical Findings and Treatment Response

Of these patients, 33 (30%) were classified in the active stage of the disease (a Mayo score of 2 or 3), while 77 (70%) were in remission (a Mayo score of 0 or 1). The positivity rate of *D. fragilis* did not differ significantly between patients in the active stage (*n* = 2, 6.1%) and those in remission (*n* = 10, 13%) (*p* > 0.05). Five of the patients (4.5%) were newly diagnosed with UC, whereas the remaining had been formerly diagnosed (varying from 1 month to 25 years) and were under follow-up. The mean disease duration of *D. fragilis*-positive patients was 5.5 ± 4.2 years, which was not significantly different from the mean duration of *D. fragilis*-negative patients (5.4 ± 3.4 years; *t* = 0.083; *p* > 0.05). Similarly, the mean BMI of patients was not different between two groups (infected: 26.2 ± 3.4; non-infected: 26.1 ± 3.6; *t* = 0.094; *p* > 0.05). The treatment response of UC patients and *D. fragilis* positivity was not different between *D. fragilis*-positive and -negative groups (*p* > 0.05). Half of the *D. fragilis*-infected patients had a healthy BMI, while 46.9% of non-infected patients were classified as overweight. In the infected group, the most common UC phenotype was left-sided colitis (50%), followed by proctitis (25%) and extensive colitis (25%). Likewise, in the non-infected group, left-sided colitis was the most common phenotype (57.1%). The most commonly used therapy in both groups was 5-ASA, with an overall frequency of 45.5%. Statistical comparisons for BMI groups, treatment regimens, and UC phenotypes were not performed because the insufficient sample sizes hindered the reliability of the results. In addition, Fisher’s exact test showed no significant association (*p* > 0.05) between *D. fragilis* positivity and any of the symptoms (Table 1). The overall presentation of *D. fragilis*-positive patients is given in Table 2. Details and comparisons of the laboratory findings are presented in Table 3.

## 4. Discussion

*Dientamoeba fragilis* is a common eukaryotic inhabitant of the human gastrointestinal tract. The interest in *D. fragilis* research has been growing in recent years, particularly focusing on its potential role in the development of gastrointestinal disorders [29]. In the present study, *D. fragilis* was detected in 12 of the 110 UC patients (10.9%) using the PCR method. The reported frequencies of *D. fragilis* were 28.4% and 16% in the same city [27,30]. An equal number of pinworm-positive and -negative samples were included in the first study. Samples for the second study were sourced from a routine diagnostic parasitology laboratory. In a larger cross-sectional study conducted in Izmir, a neighbouring city of Aydin, *D. fragilis* was detected in 12.0% of faecal samples from 490 patients with gastrointestinal complaints [31]. All the isolates in the present study were classified as genotype 1, the predominant genotype on a global scale. This finding was consistent with previous reports from Australia, Italy, Turkey, Brazil, and the Czech Republic, which suggested a highly clonal distribution of *D. fragilis* [10,11,32,33,34].

A review of the literature revealed several findings on the frequency of *D. fragilis* in UC patients, 6.1% in Turkey and 19.5% in Denmark [35,36]. Some early case reports attributed invasive ulcerating processes to *D. fragilis* infection [37]. They found numerous *D. fragilis* in the mucosal sample of a female patient with multiple ulcers and acute and chronic inflammation on rectal biopsy. Although not statistically significant, we found that the *D. fragilis* positivity rate was higher in patients in remission (13%) than in those in the active stage (6.1%). The pathogenicity of *D. fragilis* remains hypothetical and highly debated, and this finding may indicate that its presence is not associated with active mucosal inflammation in UC. It might instead indicate asymptomatic colonisation, especially in patients with stable disease. A previous study reported that, although *D. fragilis* did not invade the colonic mucosa in BALB/c mice, high-dose infections induced active colitis characterised by a marked infiltration of inflammatory cells, including eosinophils, neutrophils, and lymphocytes, within the intestinal wall [22]. Observational studies in humans evaluating UC stages have reported conflicting findings; one found that *D. fragilis* colonisation was lower in active UC (5%) compared to remission (33%) [35]. The other study did not find a correlation, reporting almost an equal distribution [36]. Overall, those studies suffered from the low number of samples in both groups. Cohort studies with large sample sizes are necessary to provide further clarity about the possible involvement of *D. fragilis* in UC pathogenesis and disease activity.

One of the primary concerns among physicians is whether *D. fragilis* should be treated, particularly given its potential association with clinical findings. Clinical-based studies have mostly compared the symptoms between *D. fragilis*-positive and -negative cases [16,17]. In the present study, clinical findings were recorded based on clinic examinations by the gastroenterologists rather than self-declarations or questionnaires. No statistically significant correlation was detected in the present study between *D. fragilis* positivity and symptoms. Many studies have reported that *D. fragilis* was related to symptoms, particularly abdominal pain, and acute or recurrent diarrhoea [16,18,38,39,40]. In addition, the faecal clearance of *D. fragilis* was associated with the resolution of clinical findings in many studies, particularly in adult patients [18,29]. However, *D. fragilis* is a common protozoan in asymptomatic individuals and no relationship to symptomatic infection has been reported [30,41,42,43,44,45]. In addition, a study from Turkey reported almost four times higher infection rates in the asymptomatic group compared to the symptomatic group: 26.9% and 8.2%, respectively [46]. The pathogenesis of *D. fragilis* has long been a subject of debate, with parasite load proposed as a potential contributing factor [47]. Studies on the genotype-based differentiation of virulent and non-virulent strains have not offered satisfactory findings yet [1]. The symptoms among the twelve positive patients in our study were abdominal/pelvic pain (three patients), diarrhoea (one patient), rectal bleeding (one patient), and fatigue (one patient). Due to the overlapping symptoms associated with UC, it is difficult to distinguish which symptoms were attributable specifically to *D. fragilis*.

The laboratory findings, including sedimentation rate, C-reactive protein, AST, ALT, total leukocyte count, neutrophils, and eosinophils, showed no statistically significant relation to *D. fragilis*. Current knowledge about the changes in laboratory parameters in *D. fragilis* infection is highly limited. It was reported that none of the blood parameters differed significantly between the 36 *D. fragilis*-positive and 72 *D. fragilis*-negative cases, except for CRP, interestingly, which was slightly lower in the *D. fragilis*-infected group [45]. Similarly, a retrospective analysis of 30 *D. fragilis*-positive cases found no pathological values in blood tests, including white blood cells, haemoglobin, and CRP [48].

This study had several limitations due to its cross-sectional design. First, the number of *D. fragilis*-positive UC patients was relatively small, which may have reduced the statistical power to detect possible significant associations between *D. fragilis* infection and clinical or laboratory findings. For example, there were only two *D. fragilis*-positive cases in the active stage of the disease. Moreover, the limited sample size restricted the use of comprehensive statistical analyses, such as multivariate modelling, to address the influence of confounding variables. Second, it was not possible to distinguish between a presumed cause and its possible effect. Third, due to the overlapping symptoms of UC and *D. fragilis* infection, it might be difficult to attribute specific symptoms to the infection. These limitations highlight the need for future longitudinal studies with larger cohorts to clarify potential correlations with disease activity, biochemical parameters, and symptomatology.

## 5. Conclusions

In conclusion, our findings suggest that *D. fragilis* likely represents either an asymptomatic coloniser in adult UC patients or has no direct impact on disease progression. Although its prevalence appeared lower in the active stage of the disease, this difference was not statistically significant. Further large-scale studies are needed to clarify the clinical significance of *D. fragilis* in the progression of ulcerative colitis.

## Figures and Tables

**Table 1:** Analysis of *D. fragilis* positivity with clinical findings and treatment response.

		*D. fragilis*		Total	Statistics ^1^ (*p* Value)
		Positive (%)	Negative		
Abdominal/pelvic pain	Yes	3 (25)	25 (25.5)	28 (25.5)	1.000
	No	9 (75)	73 (74.5)	82 (74.5)	
Iron deficiency anaemia	Yes	1 (8.3)	27 (27.6)	28 (25.5)	0.290
	No	11 (91.7)	71 (72.4)	82 (74.5)	
Rectal bleeding	Yes	1 (8.3)	30 (30.6)	31 (28.2)	0.173
	No	11 (91.7)	68 (69.4)	79 (71.8)	
Diarrhoea	Yes	0	14 (14.3)	14 (12.7)	0.357
	No	12 (100)	84 (85.7)	96 (87.3)	
Weight loss	Yes	2 (16.7)	34 (34.7)	36 (32.7)	
	No	10 (83.3)	64 (65.3)	74 (67.3)	0.178
Fatigue	Yes	0	5 (5.1)	5 (4.5)	
	No	12 (100)	93 (94.9)	105 (95.5)	1.000
Nausea/vomiting	Yes	2 (16.7)	25 (25.6)	27 (24.5)	0.848
	No	10 (83.3)	68 (69.4)	78 (70.9)	
Disease stage ^2^	Active	2 (16.7)	31 (31.6)	33 (30)	
	Remission	10 (83.3)	67 (68.4)	77 (70)	0.505
Treatment response	Yes	9 (75)	73 (74.5)	82 (74.5)	
	No	3 (25)	25 (25.5)	28 (25.5)	1.000
UC phenotype ^3^	E1	3 (25)	13 (13.3)	16 (14.5)	
	E2	6 (50)	56 (57.1)	62 (56.4)	
	E3	3 (25)	29 (29.6)	32 (29.1)	
BMI group	Healthy (18.5–24.9)	6 (50)	38 (38.8)	44 (40)	
	Overweight (25–29.9)	4 (33.3)	46 (46.9)	50 (45.5)	
	Obese (over 30)	2 (16.7)	14 (14.3)	16 (14.5)	
Treatment	5-ASA	7 (58.3)	43 (43.9)	50 (45.5)	
	5-ASA + AZA	3 (25)	27 (27.6)	30 (27.3)	
	5-ASA + BA	1 (8.3)	6 (6.1)	7 (6.4)	
	5-ASA + CS	0	3 (3.1)	3 (2.7)	
	5-ASA + AZA + BA	1 (8.3)	17 (17.3)	18 (16.4)	
	5-ASA + AZA + CS	0	2 (2)	2 (1.8)	

^1^ Fisher’s exact test; not applied to UC phenotype, BMI, and treatment variables because the contingency tables contained more than four cells. ^2^ Disease activity classification: active refers to patients with a Mayo score of 2 or 3; remission refers to those with a Mayo score of 0 or 1. ^3^ UC phenotypes: E1 is proctitis, E2 is left-sided colitis, and E3 is extensive colitis. UC: ulcerative colitis; BMI: body mass index; 5-ASA (5-aminosalicylic acid); AZA: azathioprine; BA: biological agent; CS: corticosteroid.

**Table 2:** Presentation of *D. fragilis*-positive UC patients.

No.	Age, Gender	UC Phenotype ^1^	Mayo Score ^2^	Duration (Year)	Treatment	Treatment Response	Clinical Findings
1	31, F	E3	2	<1	5-ASA + AZA	No	Abdominal/pelvic pain, diarrhoea, fatigue
2	32, M	E2	2	2	5-ASA + AZA	No	Rectal bleeding
3	41, M	E2	1	3	5-ASA + AZA + BA	Yes	None
4	51, M	E2	1	5	5-ASA	Yes	None
5	52, M	E1	0	2	5-ASA	Yes	None
6	54, M	E1	0	9	5-ASA	Yes	Fatigue
7	55, F	E1	0	2	5-ASA	Yes	None
8	66, M	E3	0	3	5-ASA	Yes	None
9	73, M	E3	1	10	5-ASA + AZA	Yes	Abdominal/pelvic pain
10	60, M	E2	1	14	5-ASA	Yes	None
11	63, M	E2	0	8	5-ASA	Yes	Abdominal/pelvic pain, fatigue
12	76, M	E3	0	8	5-ASA	Yes	None

^1^ UC phenotypes: E1 is proctitis, E2 is left-sided colitis, and E3 is extensive colitis. ^2^ Mayo score: 2 or 3 indicates active disease; 0 or 1 indicates remission. M: male; F: female; 5-ASA: 5-aminosalicylic acid (mesalazine); AZA: azathioprine; BA: biological agent; UC: ulcerative colitis.

**Table 3:** Analysis of *D. fragilis* positivity and laboratory findings ^1^.

		*D. fragilis*		Total	Statistics ^2^ (*p* Value)
		Positive (%)	Negative		
ESR ^3^	High	2 (16.7)	22 (26.2)	24 (25)	0.724
	Normal	10 (83.3)	62 (73.8)	72 (75)	
C-reactive protein	High	1(8.3)	15 (15.3)	16 (14.5)	0.518
	Normal	11 (91.7)	83 (84.7)	94 (85.5)	
AST	High	0	9 (9.2)	9 (8.2)	
	Normal	12 (100)	89 (96.8)	101 (91.8)	0.593
ALT	High	0	3 (3.1)	3 (2.7)	
	Normal	12 (100)	95 (96.9)	107 (97.3)	1.000
Total leucocytes	High	0	18 (18.4)	18 (16.4)	
	Normal	12 (100)	76 (77.6)	88 (80)	
	Low	0	4 (4.1)	4 (3.6)	
Neutrophils	High	1 (8.3)	26 (26.5)	27 (24.5)	
	Normal	11 (91.7)	72 (73.5)	83 (75.5)	0.287
Eosinophils	High	2 (16.7)	28 (28.6)	30 (27.3)	
	Normal	10 (83.3)	70 (71.4)	80 (72.7)	0.506
IDA	Yes	1 (8.3)	31 (31.6)	32 (29.1)	
	No	11 (91.7)	67 (68.4)	78 (70.9)	0.174

^1^ Reference ranges for ESR (males < 50: 0–15 mm/h; males ≥ 50: 0–20 mm/h; females < 50: 0–20 mm/h; females ≥ 50: 0–30 mm/h), CRP cut-off: 5 mg/L, liver enzymes (AST: 5–34 U/L; ALT: 0–55 U/L), and haematological parameters (TLC: 4–10 × 10^3^/µL; neutrophils: 1.5–6 × 10^3^/µL; eosinophils: 0.04–0.36 × 10^3^/µL). ^2^ Fisher’s exact test; not applied to total leukocytes because the contingency tables contained more than four cells. ^3^ ESR data were unavailable for 14 patients; thus, the analysis included 96 cases. IDA: iron deficiency anaemia.

## Data Availability

The data that support the findings of this study are available from the corresponding author upon reasonable request.

## Abbreviations

The following abbreviations are used in this manuscript:

UC	Ulcerative colitis
SSU-rRNA	Small Subunit Ribosomal RNA
BLAST	Basic Local Alignment Tool
ESR	Erythrocyte Sedimentation Rate
AST	Aspartate Aminotransferase
ALT	Alanine Aminotransferase
TLC	Total Leukocyte Count
